# Supplementary material for: I like you better when you are coherent. Narrating autobiographical memories in a coherent manner has a positive impact on listeners’ social evaluations
Source: PLoS One. 2020 Apr 30;15(4):e0232214. doi: 10.1371/journal.pone.0232214 (PMC7192457; doi:10.1371/journal.pone.0232214)
Supplement: S1 Appendix — (DOCX) [file pone.0232214.s001.docx]

 **S1 Appendix. Scoring criteria for the Narrative Coherence Coding Scheme
(Adopted from Reese et al., 2011, p. 436)**
