# Supplementary material for: I like you better when you are coherent. Narrating autobiographical memories in a coherent manner has a positive impact on listeners’ social evaluations
Source: PLoS One. 2020 Apr 30;15(4):e0232214. doi: 10.1371/journal.pone.0232214 (PMC7192457; doi:10.1371/journal.pone.0232214)
Supplement: S2 Appendix — (DOCX) [file pone.0232214.s002.docx]

**S2 Appendix. Narratives (Translated from Dutch)**

**Story 1: Coherent Positive**

One of the nicest memories I can look back on is the moment I graduated from high school. The proclamation was on a sunny day in June, in the reception hall of the school. I had been looking forward to this moment for so long. A month before, my mum and I had been going shopping in Antwerp to find a dress for me to wear, and that day really felt like quality time between the two of us. We specially planned a day to do so and had a lot of fun to make the entire outfit complete, with shoes and a bag included! On the day of the proclamation, I was just so happy. My friends, the teachers and the parents of the students, really everyone was in a good mood. We went down memory lane of the past 6 years, like how we came in as nervous freshmen, and eventually grew into adults with a broader vision on the world. People talked and laughed at the reception with some drinks and a bite to eat. Inspirational texts were read during the official part of the proclamation and I was also able to give a speech in the name of the graduating students. When doing so, I saw my mum and dad gloating from the audience. After my speech, they told me how proud they were of me, and that gave me a lot of fulfillment for all the hard work I did the past years to achieve this. I felt full of motivation to pursue higher studies and expand my own life. Eventually getting a taste of the freedom that was waiting for me at the University of Leuven. I often think back of this moment when I am currently looking for motivation to study and put my best effort in what I started.

**Story 2: Incoherent Positive**

We were walking and then he took my hand. It was full of dark green pine trees and the sun was shining from above. We know each other from that one vacation and that was really a long trip. A cold breeze but a wonderfully fresh smell. I looked into his deep brown eyes and he also was no longer able to keep his eyes off of me. Yes, it was quite a thing! I know I want to spend the rest of my life with him, he makes me feel like, yeah, I really don’t know how. We go on so many nice adventures, like that one time in the amusement park or at the see. But we can also just be together without saying anything, like in a long car drive. He put his hand behind my head and moved his head closer towards me. Everything around us disappeared and it was only the two of us in that moment. When I am with his family, I really feel, yes.. I can even get along with his mother, so not the usual mother-in-law effect haha! Yes, it’s really.. We also talked a lot on the bus afterwards and had a great time. When I look at him, there is a lot going through my mind. In the amusement park, it was so special too. My first kiss in the forest was really like in the movies. I just cannot describe what went through my mind that moment. Yes, it was a holiday to remember! I already had a feeling that there was more going on, so it was not due to the heath that I felt that way! But then in the forest it was rather chilly, so I was happy there were two of us.

**Story 3: Coherent Negative**

On May 12^th^ 2015, a friend of mine committed suicide. That morning, I went to school like other days, but when I arrived there, I quickly noticed that the atmosphere in the classroom was very heavy. Even before the first hour started, the school principal walked into our classroom. I really could not believe it when he said that Tom had left us. I really did not want to believe it. For years, I had been taking art class with Tom and over time, we had become really good friends, which was so nice. The moment I realized I would never see him again, I had so many questions still. I could not think clearly anymore, because I was consumed with grief. That day at school was horrible, all the rumors in the hallway, people who made up stuff, probably just to give themselves peace of mind. That night at home, I burst into tears in my bedroom, I felt so guilty. Why had I not been noticing anything? Why had I not been taking action? Maybe he was still here then. The same weekend, I cycled to Tom’s house. Beforehand, I sent his sister a text message to ask if I could stop by for a while, and she replied that she would indeed like me to come over. I had the feeling that I had to make up for something, at least offer support to Tom’s sister and parents. It felt good to talk with them that evening, we found support in each other’s sadness and in reminiscing about Tom. That whole time period has really changed me as a person. I will never forget this and I am still affected by it. On certain moments, I still feel sad about what happened.

**Story 4: Incoherent Negative**

I was crying at a friend’s house and really could not believe it. It was already so late. I still had not… yeah, I can hardly talk about it. He had not brought the car back on time and had been drinking again. Every now and then, I had been picking up certain things, but I just could not listen to it. I was having breakfast and my dad came stumbling in. He was mumbling a lot of things, that he would never set foot here again, that I would not see him very often in the future. I had already heard that doors were slammed and they yelled at each other, that one time when we came back from grandma for example. When he had a cup of coffee and took place across from me, I could smell him really clearly. My mum was of course furious and could not get to work. My sister missed a lot of dance classes because nobody could drive her there. They both did not keep up their appearance anymore. She did tell me once that they were growing apart. I remember mum dressing up on a certain Saturday night to go out. I was so to speak not able to understand what was going on, so she didn’t feel like explaining it to me. My sister did not have an outlet anymore and stayed here a lot. I asked if there were other people involved, but really never got an answer to that. They argued about literally everything and anything, both small issues as well as more important topics. People asked me where they were and I did not even know how to respond to that. I did not perform anymore in art class and got a lot of comments from those people. I was not doing anything good anymore. My friend could not comfort me and I just kept on crying the whole night.
