# Supplementary material for: I like you better when you are coherent. Narrating autobiographical memories in a coherent manner has a positive impact on listeners’ social evaluations
Source: PLoS One. 2020 Apr 30;15(4):e0232214. doi: 10.1371/journal.pone.0232214 (PMC7192457; doi:10.1371/journal.pone.0232214)
Supplement: S3 Appendix — (DOCX) [file pone.0232214.s003.docx]

**S3 Appendix. Full questionnaires after each narrative (Translated from Dutch)**

**Willingness***Absolutely not – Probably not – Rather not – Rather yes – Probably yes – Absolutely yes*To what extent are you willing to…
-Meet the other?
-Seek advice from the other?
-Sit on the bus with the other?
-Share an apartment with the other?
-Invite the other to your home?
-Approve if the other married a relative?
-Work with the other?
-Admit the other to your circle of friends?

**Socio-Emotional Reactions***Absolutely not – A little bit – Rather not – Rather yes – Quite a lot – Absolutely yes*
-How confusing was the story of the other?
-How pleasant was the way in which the other narrates?
-How strange do you find the other as a person?
-How well can you empathize with the other’s situation?
-How clear do you find the perspective of the other?
-To what extent do you feel close with the other?
-To what extent do you trust the other?
-To what extent do you think the other opened up to you?
-To what extent do you think the other expressed emotion?

**Feelings other***Absolutely not – A little bit – Rather not – Rather yes – Quite a lot – Absolutely ye*s
-How many positive feelings do you have for the other at the moment?
-How many negative feelings do you have for the other at the moment?

**Feelings self***Absolutely not – A little bit – Rather not – Rather yes – Quite a lot – Absolutely yes*
-How many positive feelings do you have yourself at the moment?

-How many negative feelings do you experience yourself at the moment?

**Social support: emotional***Absolutely not – Probably not – Rather not – Rather yes – Probably yes – Absolutely yes*
-I would be there to listen to her problems
-I would find ways to cheer her up when she is feeling sad
-I would try to comfort her when necessary

**Social support: instrumental***Absolutely not – Probably not – Rather not – Rather yes – Probably yes – Absolutely yes*
-I would help her when she is too busy to get everything done
-I would help her with her responsibilities, when she is unable to fulfill these

**Open question: explorative^[[1]](#footnote-1)^**
-How did you experience the way in which the other talks? What did you or did you not like? (Min. 5 sentences). Please not any other remarks you might have here.

1. **Open question**We administered the open question because of explorative reasons. Coding was done by an independent rater, who read through all the answers of the participants and categorized each response within one of three 3 categories. The first category consisted of participants who did not mention anything about narrative coherence or a coherence-related constructs (e.g. structure, cohesion, consistency). This category pertained to only 9 people (9.37%). The second category included the largest group of participants (*n* = 58, 61.46%). They did mention narrative coherence or something similar in their answer (e.g. ‘the story was incoherent and all over the place, which made it less clear’). Finally, the third category included those participants who not only mentioned narrative coherence, but also referred something to dimensions of the concept (eg. chronology: ‘the story was not told in chronological order, and this made it hard to follow’). This category consisted of 28 people (29.17%). However, this distribution did not allow us to perform further analyses, since the group that did not mention narrative coherence was too small to do so (n=9). [↑](#footnote-ref-1)
