# Supplementary material for: I like you better when you are coherent. Narrating autobiographical memories in a coherent manner has a positive impact on listeners’ social evaluations
Source: PLoS One. 2020 Apr 30;15(4):e0232214. doi: 10.1371/journal.pone.0232214 (PMC7192457; doi:10.1371/journal.pone.0232214)
Supplement: Attachment — Submitted filename: Response to reviewers_Vanaken, Bijttebier, & Hermans (2019).docx [file pone.0232214.s004.docx]

Response to reviewers

Comments
R: Replies
*M: Manuscript (L: Lines)*

 **Reviewer #1**: Overview:
In this manuscript, the authors sought to examine how memory coherence impacts socioemotional reactions in listeners. The authors use a novel (and very interesting!) methodology and found that coherent autobiographical memories were associated with empathy, trust, and increased support within the listener. This is an important topic further examining the phenomenological characteristics of autobiographical memory. Despite the work’s strengths, the manuscript needs to be streamlined. The writing can be unclear and difficult to follow, and more context/clarity is needed before this manuscript would be suitable for publication.
R: We would like to thank the reviewer for considering the topic to be important and clinically relevant as well as for the time invested in the review of this manuscript. Please find below our point-by-point replies (R) to the comments and a revised version of the *manuscript (M)*. We hope that our revision meets your expectations. We remain willing to address any further issues, questions or suggestions you may have. We have streamlined the manuscript, by leaving out and integrating parts, we hope to have better explained the concepts and rationale of this study.


Major Points:
Introduction
1. P. 4, Lines 62-69: this paragraph feels out of place. Coyne’s interactional model of depression is not described in detail, thus making it hard to understand the link between the model and how it applies to your study. Consider making a stronger argument for why this model is important for your study. At the very least, an overview of this model should be provided.

R1: We agree with the reviewer that Coyne’s interactional model of depression is not described sufficiently in the manuscript. We provide here a more extensive explanation of the rationale behind it and the link between the model and our study.
 Already in 1976, Coyne highlighted the major role that dysfunctional social behavior can play in maintaining depression. He describes in his *interactional model of depression* (Coyne, 1976a) how depressed persons and their surroundings get embedded in an increasingly negative spiral of social interactions. The depressed person expresses his/her negative thoughts, feelings and memories in such a way that feelings of guilt, hostility and annoyance are aroused in others around them. Then, in an attempt to reduce the expression of the depressive symptoms and to suppress those aversive feelings, the others react with nongenuine support and reassurance towards the depressed person. Since over time, the depressed person increases his/her seek for help and attention in a more compelling way, the others also start to reject and avoid the depressed person, which cause discrepancies between their actual behavior and the nongenuine support to become more striking. This is seen by the depressed person as a proof of their expectation that they are indeed not socially accepted and that social interactions turn out negatively. Wanting to secure their social support and to get comforted by others (need to belong; Baumeister & Leary, 1995), they display more symptoms, leading to a more negative spiral of social interactions over time.
 To present this perspective in a way that would not be too lengthy, we now added the following paragraph to the manuscript. We believe that this indeed clarifies the relation to our study. We thank the reviewer for this comment.
*M 1: (L. 80-92):* *In this process of storing, retrieving and sharing autobiographical memories, individual differences (e.g. accuracy, specificity, emotional tone) have been observed, both quantitatively as well as qualitatively, that are able to impact the extent to which the social function is served and consequently our well-being (14–16). One example can be found in the interactional model of depression, in which Coyne highlighted the major role that dysfunctional social behaviour can play in maintaining psychopathology (17). In his research, depressed persons expressed their negative thoughts, feelings and memories in such a hopeless and self-blaming way that feelings of guilt, hostility and annoyance were aroused in others around them. This led the patients and their surroundings to get embedded in an increasingly negative spiral of social interactions, causing social rejection over time, which is a major factor in maintaining depressive symptoms (18). This study is seen as important and relevant here since it was one of the first to show how characteristics of communication or narrative styles can impact social behavior and even the social maintenance of symptoms of psychopathology.*

2. P. 5: Overall, I think a stronger argument is needed to support your claim about memory incoherence possibly “causing social rejection.” I’m not sure how revealing one incoherent memory is enough to lead to social rejection. Moreover, I think it is also important to strengthen the link between memory coherence and well-being. Sure, the listener can become frustrated or confused if the memory lacks coherence, but why would one incoherent memory necessarily cause social rejection?
R2: We believe that coherence can be seen as a narrative style, that remains relatively stable over time. The consistency and stability of narrative coherence is not only evident from studies we are currently carrying out ourselves, but also from a study by Waters and colleagues (2018), who illustrate that the ways in which we tell about autobiographical events reflect a stable personal style. In our own study, however, due to the experimental nature of the design, we focused on one timepoint to measure social reactions to narrative coherence. Results showed that already being incoherent at one moment in time, caused negative social reactions. This finding strengthens the idea that being incoherent time after time, over and over again, will only increase and worsen these negative social reactions. So, we argue that even though it is already evident from revealing one incoherent memory, the negative spiral of social interactions will develop over time, causing social rejection after an individual’s incoherent narrative style gets more and more negatively reacted upon. With this in mind, the link between social support and well-being follows, which is explained in the manuscript.
*M2 (L. 120-129): In this study specifically, we will focus on the effect that memory coherence of a speaker has on social responses of a listener. It might be the case that when someone is not coherent in the sharing of personal memories, this will disturb the social function of autobiographical memory. We believe that memory coherence is part of a narrative style that remains relatively stable and consistent within individuals over time and over situations (25). We suggest that narrating incoherently can cause negative feelings (e.g. annoyance or confusion) in the listener, and that being incoherent over and over again could set in motion a negative spiral of social interactions, possibly causing social rejection with time. This means that social support could diminish when the speaker remains incoherent, which then may impact his/her psychological well-being in a negative way (7,9,10), especially in the face of adversity or trauma (risk factor for psychopathology). Similarly, telling a coherent story could be reinforced by receiving positive social feedback and support, nurturing social bonds and satisfying the need to belong over time, which could then improve well-being (protective factor for psychopathology).*

3. See points below with regard to specific issues with the information provided in the introduction. The writing needs work, the sentences should be revised to be easier to follow and understand. Overall, you should streamline the introduction and be clear about your description of prior work.
Thank you. We have provided more detailed answers to the specific issues below. We have also revised the introduction to make it easier to follow and understand.

Method
4. P. 7: More information is needed on the characteristics of the sample. Do you have information on race/ethnicity, highest level of education, etc.?
R4: All participants were Belgian, having Dutch as their mother tongue or actively speaking it. All of them finished high school, most of them were university undergraduate students (although we did not measure this, it is to be assumed looking at previous studies with similar recruitment strategies) with a mean age of 21 years old.
*M4 (L. 168-173): A total of 96 adults between the ages of 19 and 40 (M = 21.06, SD = 3.17) participated in the study, of which 84 were female and 12 were male. Our sample in this study was very homogeneous, consisting of mostly young white female students, with only a couple of outliers of people higher than 25 years in age. All of them were Belgian and indicated Dutch as their mother tongue or indicated actively speaking it. Participants signed up via the Experiment Management System (EMS) of the university, so most of them were university students.*

5. P. 7: Explain the process of narrative development. Were these narratives adapted from prior work? If not, how were they formed? How many individuals were involved in coding and were they blind to the conditions?
R5: The narratives were created by the authors, based on themes that we know are very common and representative for this sample of participants. We wrote the stories, based on our extensive experience in collecting and coding hundreds of narratives in similar samples. Starting from these 4 positive (graduation, falling in love, birthday party, travelling) and 4 negative (suicide of a friend, divorce of parents, passing away of a grandparent, end of a relationship) common themes, we wrote a coherent and an incoherent story on each theme. Subsequently 2 colleagues that were blind to the conditions, scored these narratives for coherence. Based on their ratings of the created narratives, all coherent stories received a score of 9, all incoherent stories received a score of 3. Afterwards, the 16 stories were evaluated on emotional valence by 7 new independent raters. Based on those scores, we selected the four final narratives that matched on coherence and valence, to ensure an even manipulation of our variables. We changed the manuscript as follows to provide this further information.
*M5 (L. 181 – 188): We created 16 narratives (in Dutch) based on themes that are very common in this sample and representative for self-reported events with high emotional impact.* *We wrote the stories, based on our extensive experience in collecting and coding hundreds of narratives in similar samples. We used 4 positive (graduation, falling in love, birthday party, travelling) and 4 negative themes (suicide of a friend, divorce of parents, passing away of a grandparent, end of a relationship), about which we wrote a coherent and an incoherent story each. Subsequently, 2 colleagues specialized in the field independently and blind for condition coded these 16 narratives for coherence using the Narrative Coherence Coding Scheme (NCCS; (20)).
(L. 233-241): We selected the four final narratives based on their scores for coherence and emotional valence (see S1 Appendix for full stories). We made sure to select the stories that matched as closely as possible on these scores to ensure a strict manipulation of our variables of interest. Narrative 1 was a positive (M = 4.14, SD = .69) coherent (Context = 3, Chronology = 3, Theme = 3) story about graduating from high school. Narrative 2 was a positive (M = 4.14, SD = 1.07) incoherent (Context = 1, Chronology = 1, Theme = 1) story about falling in love. Narrative 3 was a negative (M = -4.14, SD = .90) coherent (Context = 3, Chronology = 3, Theme = 3) story about the suicide of a friend. Narrative 4 was a negative (M = -4.00, SD = .82) incoherent (Context = 1, Chronology = 1, Theme = 1) story about the divorce of parents.*

6. P. 7, lines 148-149: You mention that narratives were coded as either coherent or incoherent, please make a separate sentence and describe the scoring procedure, what is included in the parentheses currently is not straightforward and I found myself reading the sentence over a few times to figure out what each category and number was referring to. What are examples of each of these categories and explain how points for each category were awarded.
R6: We apologize for the unclarities and provide some more extensive explanations hereby. To score the narratives, we followed the procedure of Reese and colleagues (2011), which is the Narrative Coherence Coding Scheme (NCCS). According to her, and in line with the literature in this domain, a coherent personal narrative is defined as one that makes sense to a naive listener, not just in terms of understanding when, where and what event took place, but also with respect to understanding the meaning of that event (Reese et al., 2011). Coherence has found to be a multidimensional characteristic of memories, that entails a contextual dimension (are the where and when of the event specified?), a chronological dimension (is the story told in chronological order?) and last but not least a thematic dimension (does the narrator have insight in the experience, does he link the event with other events in the past or possible future events, does he come to a resolution or does he reach closure?) (Reese et al., 2011). This coding system evaluates narratives on these 3 separate dimensions (score 0-3) that are summed up to entail total memory coherence (score 0-9). In a first dimension of context, we look at how the narrator orients the event in time and space. In second dimension of chronology, we evaluate whether the narrator can describe the components of the events along a logical timeline. In a third dimension, we investigate whether the narrator can maintain and elaborate emotionally on a topic, if he/she can come to a resolution or is able to reach closure. We added this in the manuscript as well and provided examples of every score for each dimension.
*M6 (L. 181-232): We created 16 narratives (in Dutch) based on themes that are very common in this sample and representative for self-reported events with high emotional impact.* *We wrote the stories, based on our extensive experience in collecting and coding hundreds of narratives in similar samples. We used 4 positive (graduation, falling in love, birthday party, travelling) and 4 negative themes (suicide of a friend, divorce of parents, passing away of a grandparent, end of a relationship), about which we wrote a coherent and an incoherent story each. Subsequently, 2 colleagues specialized in the field independently and blind for condition coded these 16 narratives for coherence using the Narrative Coherence Coding Scheme (NCCS; (20)). This coding system evaluates narratives on 3 separate dimensions (score 0-3) that are summed up to entail total memory coherence (score 0-9). Low scores on these dimension indicate incoherent narratives, whereas higher scores show a more coherently constructed narrative. In a first dimension of context, we look at how the narrator orients the event in time and space. If the narrator does not provide any information about time or place, score 0 is assigned. If there is partial information, meaning that he describes only the time in which or the location where the event took place, at any level of specificity, he receives score 1 (specific time e.g.: when I was 7 years old, nonspecific time e.g.: when I was a child, nonspecific place e.g.: when I was abroad, specific place e.g.: at my grandmother’s house). A score of 2 is assigned when both time and location are given, but no more than one dimension is specific. When he mentions time as well as location both specifically, a score of 3 is given. In a second dimension of chronology, we evaluate whether the narrator can describe the components of the events along a (chrono)logical timeline. If the narrator describes less than 3 actions of which the total event consisted (very short narratives like: when my mother passed away), a score of 0 is assigned. If the narrator describes more than 3 actions but fewer than half can be ordered on a timeline by a naïve listener, he receives score 1. When more than half of the actions can be ordered on a timeline by a naïve listener, score 2 is assigned. Score 3 is given when almost all actions can be ordered on a timeline and the narrator uses words (e.g.: right before, after an hour, the next day) to mark the temporal order of the actions. In a third dimension, we investigate whether the narrator can maintain and elaborate emotionally on a topic, if he/she can come to a resolution or is able to reach closure. Score 0 is given for narratives that are substantially off topic or are difficult to be defined in terms of a certain theme (possible themes could be e.g. the loss of a family member, a car accident, marriage). If the topic is identifiable, but minimally elaborated upon with personal evaluations (e.g.: I felt really sad, I was full of joy), score 1 is assigned. Score 2 is assigned when narratives are substantially developed around a theme and there are multiple personal interpretations or evaluations given. Finally, score 3 means that the narrator can take some meta-perspective on the event, and doesn’t only elaborate on it with momentary feelings or evaluations, but can also link it with other autobiographical events (e.g. that reminded me of the first time I fell in love), or can come to a resolution (e.g. that event made me realize how important family is for me) or reaches closure (e.g. I feel like in the end I was able to give the event a place and move on with life). After evaluation, the coherent stories we created received a score of 9, indicating they were very coherent and all incoherent stories received a score of 3, being very incoherent (score 9: Context = 3, Chronology = 3, Theme = 3; score 3: Context = 1, Chronology = 1, Theme = 1). Then, these 16 stories were evaluated on emotional valence,* *which means the extent to which they were positive or negative, by 7 new independent raters. They received the instructions to read the stories closely and after each one indicate how positive or negative they consider the story to be on a scale from -5 (extremely negative) to +5 (extremely positive). We adhered to the literature here in the sense that narrative coherence is assessed by summing the score of coherence of a very positive and a very negative event. Furthermore, social sharing of emotional events can indeed involve both positive and negative events. Hence, it was important for us to investigate whether coherence of the speaker would have an overruling effect on valence in influencing reactions of the listener (for example: even though something positive is shared, it could still be rated to be unpleasant, because it was incoherently narrated upon)*

7. P. 8, lines 157-163: With regard to my point above, without context of the scoring procedure, I’m not sure what the information presented in this section means. I would suggest creating a table to organize this information in a more coherent fashion.
R7: Thank you for your suggestion, we created a table to give an overview of the information explained in the text.
M7: Table 1. Overview of scores of narratives on coherence and valence

|  | Topic | Coherence rating (0-9) | Valence rating (-5 to +5) |
| --- | --- | --- | --- |
| Narrative 1 | Graduation | 9 | 4.14 |
| Narrative 2 | Falling in love | 3 | 4.14 |
| Narrative 3 | Suicide friend | 9 | -4.14 |
| Narrative 4 | Divorce parents | 3 | -4 |

8. P. 8, lines 169-170. Is there any prior work suggesting that there should be a gender match between the speaker and the listener? I do understand your justification for using female speakers as a majority of your sample consisted of women, but I am curious how incorporating male voices into this methodology would impact your results. Do you think it would? Why or why not? I would elaborate on this in the discussion.
R8: There is no evidence yet that social reactions to (in)coherence are gender-dependent. However, there is research from Alea and Bluck (2003) that states that gender impacts the level of closeness and intimacy in a conversation. Since we knew in advance that there would be a fair chance that our sample would consist mainly out of female participants, we opted to use all female voices to increase this closeness and to be able to investigate the possible decrease for this social response due to incoherence. However, looking at our data, there are no reasons to assume that there were any observable differences between men and women in their social reactions to (in)coherence, so we don’t think results would look different when incorporating male voices. However, future research should be carried out to test this hypothesis empirically.
*M8 (L. 622-636): Then, for gender, we opted to use all female voices, because we expected the main population of participants to be female, making the speakers more similar to themselves or their own friends and thus easier to empathise with (38).The fact that our sample comprised mostly females could at first sight also question the generalizability of our results. However, there are studies showing that gender differences might be smaller than we believe them to be in general (39). Nevertheless, we understand that gender differences in very specific domains can be of influence. For now, not much is known about this in the field of social reactions to narrative coherence, thus offering a topic for future research.*

9. P. 9: Provide justification for the decision for the authors to develop questionnaires versus using already established measures. Include information on the reliability of the measures in your analyses.
R9: We apologize for the unclarities in this part of the manuscript. The large majority of the questionnaires we used to measure social responses were not self-constructed and were taken over from original research. Our main set of questions (Willingness to interact & Social support) were developed by Coyne (1976b) and Shakespeare-Finch (2011) respectively. The questions concerning emotional attitudes are common practice in psychological research. Only the additional 9 items about empathy and trust were self-constructed after extensive literature review, hence we have no reliability data for them. Follow-up studies will take this limitation into account. We provided an extensive description of all material used in the methods section of this manuscript, also including whether or not the measures were already established or created ourselves.
*M9 (L. 258-295): We used questionnaires that would allow us to do an investigation of different socio-emotional responses of the listener to the speakers’ (in)coherent stories (See S2 Appendix for full questionnaires).
 We measured willingness to interact with the speaker, using a questionnaire of Coyne (18). This consisted of 8 questions, each to be answered on a 6-point Likert scale (ranging from ‘absolutely not’ to ‘absolutely yes’), giving a possible minimum score of 8 and a maximum score of 48. Questions contained, for example, the willingness to meet the other, seek advice from the other and sit on the bus with the other.
 We assessed a range of socio-emotional reactions regarding trust and empathy towards the speaker with 9 questions, each to be answered on a similar 6-point Likert scale (ranging from ‘absolutely not’ to ‘absolutely yes’), giving a possible minimum score of 9 and a maximum score of 54. Questions concerned, for example, how confusing they thought the story was, how well they can empathize with the other, to what extent they could trust the other etc. Items of this questionnaire were created by the authors after extensive literature review, to investigate as closely as possible our variables of interest.
 We assessed momentary positive and negative feelings towards the other and experiencing themselves using 4 items (How much positive feelings do you have for the other at the moment?, How much negative feelings do you have for the other at the moment?, How much positive feelings do you have yourself at the moment?, How much negative feelings do you experience yourself at the moment?). Each question was to be rated on a similar 6-point Likert scale (ranging from ‘absolutely not’ to ‘absolutely yes’), giving a minimum score of 1 and a maximum score of 6 on each of the four items.
 We measured social support with the 2-Way Social Support Scale of Shakespeare-Finch and colleagues (28), using 3 items measuring emotional support and 2 items measuring instrumental support. Emotional support assessed elements like: I would be there to listen to his/her problems, whereas instrumental support measured things as: I would help him/her when he/she is too busy to get everything done. Both were rated on the same 6-point Likert scale (ranging from ‘absolutely not’ to ‘absolutely yes’), resulting in a minimum score of 3 and a maximum score of 18 for emotional support and a minimum score of 2 and a maximum score of 12 for instrumental support.*


Results
10. Given the 19-40 age-range in the sample, did you take age into consideration as a covariate? I am curious how socioemotional reactions would differ across age, and even other demographic variables such as race, education, etc.
R10: This would indeed be a very interesting question, however not possible to investigate in this study. Our sample in this study was very homogeneous, consisting of mostly young white female students, with only a couple of outliers of people higher than 25 years in age. The subsample of relatively older ages is hence to small too conduct further analyses on, which means it would not allow us to draw conclusions for different demographics. In a follow-up study, it would be interesting to investigate how socio-emotional reactions would differ in a more heterogeneous sample.
*M10 (L169-172): Our sample in this study was very homogeneous, consisting of mostly young white female students, with only a couple of outliers of people higher than 25 years in age.*

11. Because you split up your results into sections (e.g., willingness, socioemotional reactions, etc.), I would provide more information about how these were assessed in the Method section. I appreciate that you have included the full questionnaires in the Appendix, but a few sample items and specifically outlining which socioemotional reactions were measured and how they were measured would be beneficial to your reader.
R11: Thank you for your suggestion. For completeness matters, we extended the examples in the Method section.

*M11 (L. 258-295): We used questionnaires that would allow us to do an investigation of different socio-emotional responses of the listener to the speakers’ (in)coherent stories (See S2 Appendix for full questionnaires).
 We measured willingness to interact with the speaker, using a questionnaire of Coyne (18). This consisted of 8 questions, each to be answered on a 6-point Likert scale (ranging from ‘absolutely not’ to ‘absolutely yes’), giving a possible minimum score of 8 and a maximum score of 48. Questions contained, for example, the willingness to meet the other, seek advice from the other and sit on the bus with the other.
 We assessed a range of socio-emotional reactions regarding trust and empathy towards the speaker with 9 questions, each to be answered on a similar 6-point Likert scale (ranging from ‘absolutely not’ to ‘absolutely yes’), giving a possible minimum score of 9 and a maximum score of 54. Questions concerned, for example, how confusing they thought the story was, how well they can empathize with the other, to what extent they could trust the other etc. Items of this questionnaire were created by the authors after extensive literature review, to investigate as closely as possible our variables of interest.
 We assessed momentary positive and negative feelings towards the other and experiencing themselves using 4 items (How much positive feelings do you have for the other at the moment?, How much negative feelings do you have for the other at the moment?, How much positive feelings do you have yourself at the moment?, How much negative feelings do you experience yourself at the moment?). Each question was to be rated on a similar 6-point Likert scale (ranging from ‘absolutely not’ to ‘absolutely yes’), giving a minimum score of 1 and a maximum score of 6 on each of the four items.
 We measured social support with the 2-Way Social Support Scale of Shakespeare-Finch and colleagues (28), using 3 items measuring emotional support and 2 items measuring instrumental support. Emotional support assessed elements like: I would be there to listen to his/her problems, whereas instrumental support measured things as: I would help him/her when he/she is too busy to get everything done. Both were rated on the same 6-point Likert scale (ranging from ‘absolutely not’ to ‘absolutely yes’), resulting in a minimum score of 3 and a maximum score of 18 for emotional support and a minimum score of 2 and a maximum score of 12 for instrumental support.*

12. P. 17: a correlation matrix would be helpful to include as a table to make clear which variables were included in your analysis.
R12: We investigated correlations between all social reactions and the following person variables: psychological well-being, empathy, extraversion, neuroticism, openness, conscientiousness, agreeableness, depression, stress, and anxiety. Including the full correlation matrix in the manuscript is an option that we have considered. However, because it is quite large, we decided to only report on the significant findings. We provided it here for your information. However, in case you and the editor would nevertheless believe that it would still merit including it in the final manuscript, we would of course be happy to do so.

Discussion
13. The authors do have some really interesting findings here, but the discussion should be refocused to emphasize what exactly this is adding to the literature. Spend less time on repeating the results and explain how it fits into the work that’s already been done on this topic. Relatedly, how do your findings relate back to the literature that was cited in the introduction? The authors do refer back to Coyne’s model, but not enough context was provided in the introduction to fully appreciate how this links to your findings.
R13: Thank you for considering these results to be interesting. We added more information about Coyne’s model in the introduction, to make the link to our findings easier to follow. Moreover, we restructured both the introduction and the discussion to emphasize the added value to the literature. Changes were tracked in the manuscript.
*M13 (L. 605-609): This is in line with evidence that Coyne (17) found for the impact of the specific ways in which depressive patients narrate on social reactions they receive.*

Other Points:
1. Abstract: The word limit for abstracts is 300, thus I would suggest shortening this. Consider streamlining your introductory points about prior literature. Use the word count available to more clearly outline your methods and discuss the implications of your work.
R1: We shortened the abstract to fit the word limit of 300. We left out a couple of the introductory points to shift the focus on methods and implications.

2. Introduction, p. 3, lines 49-61: I would consider reorganizing the paragraph, as some of the descriptions of the prior work in this area are not clear and are hard to follow. For example, what do you mean by fictional relationship events? When you discuss the research conducted by Lyubomirsky, Sousa, and Dickerhoof, elaborate on your point that writing/talking about past negative experiences is related to better physical/mental health compared to “mere thinking about it”. How was this tested? It is unclear whether or not sharing via writing/talking about one’s experience would be beneficial if the person were to do it by oneself (e.g., writing experiences down) or in a social setting.
R2: Thank you for the comment. We left out the study of Lyubomirsky and colleagues and changed the paragraph to make it easier to follow. These fictional relationship events were non-autobiographical vignettes, so about memories that participants did not ‘own’. For example, participants received these instructions: ‘listen to a story about a vacation that another couple had together’. They were then asked to ‘tell me everything you can remember about the positive vacation that the couple had together. Tell me about where they were, what they did, and what they were thinking and feeling’.
*M2 (L. 61-65): For example, Alea and Bluck (8) found that indicators of intimacy such as warmth and closeness to others, increased after narrating about personally experienced relationship events (e.g their own vacation) as opposed to talking about non-autobiographical vignettes (e.g. another couple’s vacation).*

3. P. 3, line 56-57: Because you are interested in explaining underlying mechanisms that can explain the relationship between memory coherence and well-being, and because you hypothesize that social support plays an instrumental factor, you should discuss the mechanism behind the notion that social support enhances resilience and protects against psychopathology.
R3: There is extensive literature on the importance of a social network to enhance well-being and protect against psychopathology. The mechanism behind it concerns our evolutionary evolved need to belong (Baumeister & Leary, 1995). Since this is an implication of our results and not the main focus of the experiment, we refer to the literature in the manuscript as follows.
  *M3 (L. 57-71): The social side of the autobiographical memory has shown to be very useful (5). Research states that we share up to 90% of memories of emotional events on the same day and even within a couple of hours after experiencing them (6). Even more importantly, not only does this happen very frequently, this social sharing also has a clear underlying function. It facilitates the development and maintenance of social relationships over time and thereby fulfils our primary need to belong (7). For example, Alea and Bluck (8) found that indicators of intimacy such as warmth and closeness to others, increased after narrating about personally experienced relationship events (e.g their own vacation) as opposed to talking about non-autobiographical vignettes (e.g. another couple’s vacation). This is in line with extensive evidence that a stable social network is very valuable for ensuring physical and mental health (9,10). It has been suggested that good social support enhances resilience to stress and is a protective factor for psychopathology (11). Likewise, a lack of social support has been associated with a decrease in psychological well-being and a higher likelihood of developing feelings of loneliness, symptoms of depression (12,13). In sum, sharing personal memories allows us to develop a social network, thereby providing us with a sense of belonging, which is vital for our psychological well-being.*

4. P. 4. Line 61: You should explain further how you (and others you cite) operationalize psychological well-being.
R4: In line with the existing literature, psychological-well being is defined and operationalized in terms of the six scales of well-being developed by Ryff (1989). It measures 6 aspects of well-being and happiness, which are autonomy, environmental mastery, personal growth, positive relations with others, purpose in life, and self-acceptance. We used the shortened and related version of this instrument, called the Flourishing Scale, that has shown to be a reliable and valid measure of psychological well-being. (Ryff CD. Happiness is everything, or is it? Explorations on the meaning of psychological well-being. Journal of Personality and Social Psychology. 1989;57:1069–1081.)
*M4 (L. 298-308): To measure psychological well-being, we used the Flourishing Scale (FS) (29,30). This is a short 8-item instrument to measure psychosocial prosperity. It has good psychometric properties and is related to other psychological well-being scales (31).*

5. P. 5, line 91: Be specific as to how you are operationalizing each construct (such as social support, well-being, etc.) in your study. It is unclear what “good psychological functioning” means.
R5: Thank you for your question. We eliminated using the term ‘good psychological functioning’ and consistently used the term ‘psychological well-being’. We have provided more thorough descriptions of the concepts in the manuscript as follows.
*M5 (L. 289-295):* *We measured social support with the 2-Way Social Support Scale* *of Shakespeare-Finch and colleagues (28), which consists of 3 items measuring emotional support and 2 items measuring instrumental support. Emotional support assessed elements like: I would be there to listen to his/her problems, whereas instrumental support measured things as: I would help him/her when he/she is too busy to get everything done. Both were rated on the same 6-point Likert scale (ranging from ‘absolutely not’ to ‘absolutely yes’), resulting in a minimum score of 3 and a maximum score of 18 for emotional support and a minimum score of 2 and a maximum score of 12 for instrumental support.*
 *(L. 298-301): To measure psychological well-being, we used the Flourishing Scale (FS) (29,30). This is a short 8-item instrument to measure psychosocial prosperity. It has good psychometric properties and is related to other psychological well-being scales (31).*

6. P. 5, lines 93-95: As I was reading this, the first thing I asked myself is whether the relationship between memory incoherence and negative social interactions is seen among close peers? Family members? Does this relationship change based on the relationship between the speaker and the listener?
R6: Thank you for your interesting question. Since this is only a first exploratory study in this largely under-investigated domain, we don’t have an answer yet to the question whether and how the specifics of a relationship will influence social reactions to coherence. Most likely, the precise relationship will interact with the way coherence impacts social reactions, so we would hypothesize that the bigger social picture influences the precise effects of narrative style investigated in this study. For example, you could expect more tolerance for incoherence in close friends or family members, however this has not been empirically investigated as of yet. This was a first experimental study focused on studying social reactions to coherence in depth (experimentally, exclusion of other variables), rather than broadly (naturalistic, inclusion of other variables). We integrated this question in our manuscript as follows.
*M6 (L. 637-647): Furthermore, the interaction between speaker and listener (e.g. the specific nature of their relationship: peers, family members, romantic partners) was filtered out in our experiment, which has a large benefit of having a clearer measure of the mere causal effect of memory coherence but carries the disadvantage of being dissimilar to a real life social interaction and undermining the complexity of the issues at stake. The multifaceted nature of narratives and its relation to social interaction (e.g. biographical embedding vs absence of it, inclusion of others’ subjective perspectives vs exclusion, the extent to which the event concerned the interest of the individual and whether the participants had been through a similar situation themselves; (40)) illustrates this complexity, but goes beyond the scope of this first study. It would be very interesting to consider these variables in a follow-up study since we do not know of any evidence regarding the covariance of those variables with our measure of memory coherence.*

7. P. 6, line 109: What do incoherent narratives consist of? It is important to define and explain these important terms for your reader, especially if they have not been exposed to research on memory phenomenology. You should do the same for memory valence, especially because it was assessed in the current study.
R7: Thank you for your questions. We followed the exact procedure of Reese and colleagues (2011), which is the Narrative Coherence Coding Scheme (NCCS). According to her, and in line with the literature in this domain, a coherent personal narrative is defined as one that makes sense to a naive listener, not just in terms of understanding when, where and what event took place, but also with respect to understanding the meaning of that event (Reese et al., 2011). Coherence has found to be a multidimensional characteristic of memories, that entails a contextual dimension (are the where and when of the event specified?), a chronological dimension (is the story told in chronological order?) and last but not least a thematic dimension (does the narrator have insight in the experience, does he link the event with other events in the past or possible future events, does he come to a resolution or does he reach closure?) (Reese et al., 2011). We added more extensive explanations and examples in the manuscript as follows. Furthermore, we provided a better description of memory valence. We hope to have made these concepts more clear now.  *M7 (L. 181-231): We created 16 narratives (in Dutch) based on themes that are very common in students’ self-reported events with high emotional impact. We used 4 positive and 4 negative themes, about which we wrote a coherent and an incoherent story each. Subsequently, 2 colleagues specialized in the field independently and blind for condition coded these 16 narratives for coherence using the Narrative Coherence Coding Scheme (NCCS; (20)). This coding system evaluates narratives on 3 separate dimensions (score 0-3) that are summed up to entail total memory coherence (score 0-9). Low scores on these dimension indicate incoherent narratives, whereas higher scores show a more coherently constructed narrative. In a first dimension of context, we look at how the narrator orients the event in time and space. If the narrator does not provide any information about time or place, score 0 is assigned. If there is partial information, meaning that he describes only the time in which or the location where the event took place, at any level of specificity, he receives score 1 (specific time e.g.: when I was 7 years old, nonspecific time e.g.: when I was a child, nonspecific place e.g.: when I was abroad, specific place e.g.: at my grandmother’s house). A score of 2 is assigned when both time and location are given, but no more than one dimension is specific. When he mentions time as well as location both specifically, a score of 3 is given. In a second dimension of chronology, we evaluate whether the narrator can describe the components of the events along a (chrono)logical timeline. If the narrator describes less than 3 actions of which the total event consisted (very short narratives like: when my mother passed away), a score of 0 is assigned. If the narrator describes more than 3 actions but fewer than half can be ordered on a timeline by a naïve listener, he receives score 1. When more than half of the actions can be ordered on a timeline by a naïve listener, score 2 is assigned. Score 3 is given when almost all actions can be ordered on a timeline and the narrator uses words (e.g.: right before, after an hour, the next day) to mark the temporal order of the actions. In a third dimension, we investigate whether the narrator can maintain and elaborate emotionally on a topic, if he/she can come to a resolution or is able to reach closure. Score 0 is given for narratives that are substantially off topic or are difficult to be defined in terms of a certain theme (possible themes could be e.g. the loss of a family member, a car accident, marriage). If the topic is identifiable, but minimally elaborated upon with personal evaluations (e.g.: I felt really sad, I was full of joy), score 1 is assigned. Score 2 is assigned when narratives are substantially developed around a theme and there are multiple personal interpretations or evaluations given. Finally, score 3 means that the narrator can take some meta-perspective on the event, and doesn’t only elaborate on it with momentary feelings or evaluations, but can also link it with other autobiographical events (e.g. that reminded me of the first time I fell in love), or can come to a resolution (e.g. that event made me realize how important family is for me) or reaches closure (e.g. I feel like in the end I was able to give the event a place and move on with life). After evaluation, the coherent stories we created received a score of 9, indicating they were very coherent and all incoherent stories received a score of 3, being very incoherent (score 9: Context = 3, Chronology = 3, Theme = 3; score 3: Context = 1, Chronology = 1, Theme = 1). Then, these 16 stories were evaluated on emotional valence,* *which means the extent to which they were positive or negative, by 7 new independent raters. They received the instructions to read the stories closely and after each one indicate how positive or negative they consider the story to be on a scale from -5 (extremely negative) to +5 (extremely positive). We adhered to the literature here in the sense that narrative coherence is assessed by summing the score of coherence of a very positive and a very negative event. Furthermore, social sharing of emotional events can indeed involve both positive and negative events. Hence, it was important for us to investigate whether coherence of the speaker would have an overruling effect on valence in influencing reactions of the listener (for example: even though something positive is shared, it could still be rated to be unpleasant, because it was incoherently narrated upon).*

8. Discussion, p. 23, lines 469-471: I am not seeing the link between memory coherence and how you arrive at the conclusion that incoherent memories can elicit negative interactions in therapy specifically, given that therapists receive specialized training. Is there any evidence that supports your claim? If so, include it to strengthen the argument. If not, I would consider taking removing this and include more realistic implications for your work.
R8: Thank you for your concern regarding this part of the discussion. There is some evidence that the manner in which clients talk has an effect on the therapist, even if they have received professional training. For example, there is some work on transference and countertransference in this regard and the slowing down of therapy processes because of this. Even though therapists may be trained and would be less likely to explicitly give negative reactions, their mood could be lowered and sessions may go slower because of confusion that incoherence evokes. However, clinical studies are still to be done to investigate the precise conditions under which incoherence could disturb therapy processes and how those effects take place. (Horowitz LM, Rosenberg SE, Bartholomew K. Interpersonal problems, attachment styles, and outcome in brief dynamic psychotherapy. Journal of Consulting and Clinical Psychology. 1993;61:549–560., Martin A, Buchheim A, Berger U, Strauss B. The impact of attachment organization on potential countertransference reactions. Psychotherapy Research. 2007;17:46–58.)

*M8 (L.670-678): A first domain is that of psychotherapy and counselling, in which narration of personal experiences is central. The manner in which clients talk to their therapist could have an effect on the social or emotional reactions of the latter. Even though therapists may be professionally trained and would be less likely to explicitly give negative reactions, their mood could be lowered and sessions may go slower because of confusion or unclarity that incoherence brings with. Therapists might also be inclined to interact in a more negative way with clients that talk incoherently, might be less willing to interact with them, and to provide the same support as they would to a more coherent patient. This can be also related to research about coherence in attachment styles (41,42).*

9. P. 23-24, lines 487-488: What type of “training” would teach people to report more coherent memories?
R9: In the domain of autobiographical memory specificity, our lab has been at the forefront of developing a training aimed at improving reduced autobiographical memory specificity, named Memory Specificity Training (MeST; Raes, Williams, & Hermans, 2009), which has been proven to be effective in individuals with depressive symptomatology. Similarly, we would like to develop, in a further stage when more basic knowledge on memory coherence has been acquired and would support this opportunity, a training that targets memory coherence. This intervention could go as follows: First, participants will fill out questionnaires regarding social functioning and psychological well-being as a pre-measurement. Then, they will receive an online psycho- education session about narrative coherence, its dimensions and its implications for social bonding and mental health. In the subsequent 5 weeks, they will receive an online training to improve their narrative coherence, both for long lasting memories, as well as for daily social interactions. We could use experience sampling techniques to ask participants every other day to report a memory or a recent experience, after being given a cue word. Then, they will receive specific instructions to write (type in their phone) coherently about that memory, using enhanced narrative coherence instructions, which go over every separate dimension of coherence to evoke detailed responses. Afterwards, they will receive exercises and concrete tips to use this knowledge in their conversations to others, so that they not only write, but also talk coherently in their everyday life. Finally, after the 5-week intervention, they will fill out the questionnaires again, as a post-measurement.
*M9: (L. 717-727): Since this is only the first study investigating the domain of social reactions in relation to memory coherence, follow-up research and replication studies (e.g. looking at the different dimensions of memory coherence) are needed before developing some sort of training. This training could be carried out online, using experience sampling techniques to ask participants to report a memory or a recent experience. Using enhanced narrative coherence instructions, we could ask them to provide detailed responses to questions about each separate dimension of coherence, and in that sense teach them to create stories that are coherent for both context, chronology and theme. Afterwards, they will receive exercises and concrete tips to use this knowledge in their conversations to others, so that they not only write, but also talk coherently in their everyday life. Future studies will have to investigate whether this helps building social relationships and improving psychological well-being.*

10. Please check APA reporting guidelines. When providing means, standard deviations, p-values, t-values, etc., these symbols should be in italics.
R10: Thank you for noticing, we made the changes in the manuscript accordingly.

**Reviewer #2:** Thank you for the opportunity to read and review the paper. I provide my comments below.

1. What is unique about autobiographical memories – vs. any memories/ stories? – or, in fact, vs. any conversational content/matter – with respect to the proposed processes? Are there different/unique effects posited for autobiographical memories – versus other memories – or for any conversational matter? I would add more about this in the Introduction. Also, going forward, I might include a non-autobiographical condition to parse this out.
R1: Autobiographical memories are unique in the sense that they specifically function to serve our sense of self and our social well-being. It are also specifically our autobiographical memories that are such a very frequent topic of conversation, more so than general semantic topics. It is indeed good to notice that this effect of coherence probably goes further than only autobiographical memories and could concern a general (in)coherence trait that evokes different social reactions. However, it is to be expected that, specifically because of that social function (i.e. bonding) it serves, incoherence in autobiographical memories notably disrupts our social functioning, rather than talking incoherently about other things (since they don’t serve that social function). Furthermore, the way we define coherence, is in particular relevant for autobiographical memories, because it contains the dimension of theme. This thematic component (personal evaluations, linking to other autobiographical events, resolutions) is absent in more general semantic conversations (e.g. route descriptions, facts, knowledge).
We changed this in the manuscript accordingly.

*M1 (L. 656-669) In particular, this concerns our autobiographical memories more so than any other semantic conversational topic, since they are unique as defined by their function to serve our social well-being and sense of self. It are also specifically our autobiographical memories that are such a very frequent topic of conversation, more so than general semantic topics. It is good to notice that this effect of coherence probably goes further than only autobiographical memories and could concern a general (in)coherence trait that evokes different social reactions. However, it is to be expected that, specifically because of that social function (i.e. bonding) it serves, incoherence in autobiographical memories notably disrupts our social functioning, rather than talking incoherently about other things, since the latter doesn’t serve that social function.*

2. I apologize that I was not completely clear on one aspect of the methodology. My understanding is that there were different “coherent” and “incoherent” stories. If this is correct, I wonder why this was the case – versus creating positive and negative and coherent and incoherent versions of EACH given story. Perhaps the latter was done, though? Because I am not certain, I do not take this into account in my ratings and overall recommendation. In any case, I would encourage the authors to make sure this is very clear in the Methodology.
R2: It is indeed correct that there were coherent and incoherent stories about different themes, as we wanted it to sound like there were 4 different persons, each talking about their own different experience. Hence it was not possible to create stories about the same topic. We do argue however that it would be a good option to frame the experiment differently and use the same theme (=topic) to create more even stories that only differ in coherence. We will take this into account when designing a follow-up study.

*M5 (L. 233-241): We selected the four final narratives based on their scores for coherence and emotional valence (see S1 Appendix for full stories). We made sure to select the stories that matched as closely as possible on these scores to ensure a strict manipulation of our variables of interest. Narrative 1 was a positive (M = 4.14, SD = .69) coherent (Context = 3, Chronology = 3, Theme = 3) story about graduating from high school. Narrative 2 was a positive (M = 4.14, SD = 1.07) incoherent (Context = 1, Chronology = 1, Theme = 1) story about falling in love. Narrative 3 was a negative (M = -4.14, SD = .90) coherent (Context = 3, Chronology = 3, Theme = 3) story about the suicide of a friend. Narrative 4 was a negative (M = -4.00, SD = .82) incoherent (Context = 1, Chronology = 1, Theme = 1) story about the divorce of parents.*

3. I wonder if coherence is thought to be a reflective or formative construct. That is, are these components/dimensions interchangeable/compensatory or must they all be present (to some degree) for a story to be coherent?
R3: Thank you for your interesting question. Coherence has been found to be a multidimensional characteristic of memories, that entails a contextual dimension (are the where and when of the event specified?), a chronological dimension (is the story told in chronological order?) and last but not least a thematic dimension (does the narrator have insight in the experience, does he link the event with other events in the past or possible future events, does he come to a resolution or does he reach closure?) (Reese et al., 2011). The total score for memory coherence (0-9) is calculated by summing the scores for all 3 dimensions (0-3). The higher the score, the more coherent the story is to be found. All dimensions need to be present to get a very high score, however intermediate scores can be reached in different ways (e.g. low context, high chronology, low theme or high context, low chronology, low theme), so there is a certain compensation or interchangeability in the measure. We have included this in the manuscript as follows.

*M2 (L. 188-222): This coding system evaluates narratives on 3 separate dimensions (score 0-3) that are summed up to entail total memory coherence (score 0-9). Low scores on these dimension indicate incoherent narratives, whereas higher scores show a more coherently constructed narrative. All dimensions need to be present to get a very high score, however intermediate scores can be reached in different ways (e.g. low context, high chronology, low theme or high context, low chronology, low theme), so there is a certain compensation or interchangeability in the measure. In a first dimension of context, we look at how the narrator orients the event in time and space. If the narrator does not provide any information about time or place, score 0 is assigned. If there is partial information, meaning that he describes only the time in which or the location where the event took place, at any level of specificity, he receives score 1 (specific time e.g.: when I was 7 years old, nonspecific time e.g.: when I was a child, nonspecific place e.g.: when I was abroad, specific place e.g.: at my grandmother’s house). A score of 2 is assigned when both time and location are given, but no more than one dimension is specific. When he mentions time as well as location both specifically, a score of 3 is given. In a second dimension of chronology, we evaluate whether the narrator can describe the components of the events along a (chrono)logical timeline. If the narrator describes less than 3 actions of which the total event consisted (very short narratives like: when my mother passed away), a score of 0 is assigned. If the narrator describes more than 3 actions but fewer than half can be ordered on a timeline by a naïve listener, he receives score 1. When more than half of the actions can be ordered on a timeline by a naïve listener, score 2 is assigned. Score 3 is given when almost all actions can be ordered on a timeline and the narrator uses words (e.g.: right before, after an hour, the next day) to mark the temporal order of the actions. In a third dimension, we investigate whether the narrator can maintain and elaborate emotionally on a topic, if he/she can come to a resolution or is able to reach closure. Score 0 is given for narratives that are substantially off topic or are difficult to be defined in terms of a certain theme (possible themes could be e.g. the loss of a family member, a car accident, marriage). If the topic is identifiable, but minimally elaborated upon with personal evaluations (e.g.: I felt really sad, I was full of joy), score 1 is assigned. Score 2 is assigned when narratives are substantially developed around a theme and there are multiple personal interpretations or evaluations given. Finally, score 3 means that the narrator can take some meta-perspective on the event, and doesn’t only elaborate on it with momentary feelings or evaluations, but can also link it with other autobiographical events (e.g. that reminded me of the first time I fell in love), or can come to a resolution (e.g. that event made me realize how important family is for me) or reaches closure (e.g. I feel like in the end I was able to give the event a place and move on with life). After evaluation, the coherent stories we created received a score of 9, indicating they were very coherent and all incoherent stories received a score of 3, being very incoherent (score 9: Context = 3, Chronology = 3, Theme = 3; score 3: Context = 1, Chronology = 1, Theme = 1).*

4. Several aspects of the measurement seem potentially problematic. First, I would encourage using established (i.e., “validated) measures where possible – especially for constructs like the current ones (e.g., trust), where established measures exist. Related to this, there should be psychometric analyses/results for the measures reported in the paper (e.g., factor analyses, reliability). Finally, I believe there should be some discussion of the well-being variables in the Introduction. Well-being certainly is a complex multidimensional construct, and so attention should be given to the rationale and theory regarding the outcomes in the front end of the paper.

R4(a): We apologize for the unclarities in this part of the manuscript. The large majority of the questionnaires we used to measure social responses were not self-constructed and were literally taken over from original research. Our main set of questions (Willingness to interact & Social support) were developed by Coyne (1976b) and Shakespeare-Finch (2011) respectively. The questions concerning emotional attitudes are common practice in psychological research. Only the additional 9 items about empathy and trust were self-constructed after extensive literature review, hence we have no reliability data for them. Follow-up studies will take this limitation into account. We provided an extensive description of all material used in the methods section of this manuscript, also including whether or not the measures were already established or created ourselves.

*M9 (L. 258-295): We used questionnaires that would allow us to do an investigation of different socio-emotional responses of the listener to the speakers’ (in)coherent stories (See S2 Appendix for full questionnaires).
 We measured willingness to interact with the speaker, using a questionnaire of Coyne (18). This consisted of 8 questions, each to be answered on a 6-point Likert scale (ranging from ‘absolutely not’ to ‘absolutely yes’), giving a possible minimum score of 8 and a maximum score of 48. Questions contained, for example, the willingness to meet the other, seek advice from the other and sit on the bus with the other.
 We assessed a range of socio-emotional reactions regarding trust and empathy towards the speaker with 9 questions, each to be answered on a similar 6-point Likert scale (ranging from ‘absolutely not’ to ‘absolutely yes’), giving a possible minimum score of 9 and a maximum score of 54. Questions concerned, for example, how confusing they thought the story was, how well they can empathize with the other, to what extent they could trust the other etc. Items of this questionnaire were created by the authors after extensive literature review, to investigate as closely as possible our variables of interest.
 We assessed momentary positive and negative feelings towards the other and experiencing themselves using 4 items (How much positive feelings do you have for the other at the moment?, How much negative feelings do you have for the other at the moment?, How much positive feelings do you have yourself at the moment?, How many negative feelings do you experience yourself at the moment?). Each question was to be rated on a similar 6-point Likert scale (ranging from ‘absolutely not’ to ‘absolutely yes’), giving a minimum score of 1 and a maximum score of 6 on each of the four items.
 We measured social support with the 2-Way Social Support Scale of Shakespeare-Finch and colleagues (28), using 3 items measuring emotional support and 2 items measuring instrumental support. Emotional support assessed elements like: I would be there to listen to his/her problems, whereas instrumental support measured things as: I would help him/her when he/she is too busy to get everything done. Both were rated on the same 6-point Likert scale (ranging from ‘absolutely not’ to ‘absolutely yes’), resulting in a minimum score of 3 and a maximum score of 18 for emotional support and a minimum score of 2 and a maximum score of 12 for instrumental support.*

(b) In line with the existing literature, psychological-well being is defined and operationalized in terms of the six scales of well-being developed by Ryff (1989). It measures 6 aspects of well-being and happiness, which are autonomy, environmental mastery, personal growth, positive relations with others, purpose in life, and self-acceptance. We used the shortened and related version of this instrument, called the Flourishing Scale, that has shown to be a reliable and valid measure of psychological well-being. In this specific study, we were mainly looking at the social reactions to memory coherence, the impact on well-being is only a side-effect and implication of that, hence we are not focusing on that and refer to how it is commonly used in this domain. (Ryff CD. Happiness is everything, or is it? Explorations on the meaning of psychological well-being. Journal of Personality and Social Psychology. 1989;57:1069–1081.)
*M4(b) (L. 265-268): To measure psychological well-being, we used the Flourishing Scale (FS) (29,30). This is a short 8-item instrument to measure psychosocial prosperity. It has good psychometric properties and is related to other psychological well-being scales (31).*

5. This sentence seems tangential/unnecessary. “Relatedly, Lyubomirsky, Sousa, and Dickerhoof [9] showed that writing or talking about a past negative experience was
related to better physical and psychological health, compared to mere thinking about it.” It does not seem to support the social functioning/well-being argument being made at that point.
R5: We agree with the comment and the sentence was left out in the manuscript.

6. I strongly would encourage looking at Pennebaker’s work on autobiographical narratives and well-being. Especially relevant, his work shows that more coherent autobiographical narratives provide a sense-making function (i.e., another, non-social, mediating mechanism). So, social functioning may be ONE mediating mechanism between coherence and well-being, but it is almost certainly not the only one. See, for example,

Pennebaker, J. W., Mayne, T. J., & Francis, M. E. (1997). Linguistic predictors of adaptive bereavement. Journal of Personality and Social Psychology,72(4), 863.doi:10.1037/0022-3514.72.4.863

R6: We agree that the expressive writing literature is very interesting. The social function is indeed only ONE of different possible mechanisms, and there are likely many other ones operating between memory coherence and well-being, that can be brought back to other functions of memory. Meaning-making for example would serve the self-function of autobiographical memory, and indeed not just the social function. However, to keep the manuscript focused on verbal expression of memories, we decided to not include detail on Pennebaker’s work. We described this in the manuscript as follows.
*M6 (L. 53-56): In addition to this self-in-relation function (developing and nurturing social bonds by not only remembering but also sharing past experiences with others), there is evidence for a self-definition function (using past experiences to create a sense of self and meaning-making) and a self-regulation function (directing future behaviour based on past experiences) (2–4).
(L. 116-119): However, it is not clear yet what the precise mechanisms are that underlie the relation between autobiographical memory coherence and psychological well-being. Concerning this question, it is suggested that the earlier mentioned social function of autobiographical memory might be one of the mechanisms at stake.*

7. Given the number of DV’s assessed, I would suggest using a Bonferroni correction for all tests – not just those for the secondary variables.
R7: Thank you for this recommendation. In line with Perneger (1998), we argue that applying Bonferroni corrections for a priori hypotheses has advantages and disadvantages. One of the disadvantages is that the adjusted α is often smaller than necessary, particularly if there are quite a few tests and/or the test statistics are positively correlated (all social reactions). Therefore, this method often fails to detect real differences and implies an increase in likelihood of type II-error to occur (Lee & Lee, 2018). After weighing out the (dis)advantages, we decided to not apply this conservative correction to our dependent variables that measured social reactions, to not miss possible effects and to avoid type II-errors. We did however decide to apply them for the analyses with the person variables that were exploratory (as defined in the AsPredicted pre-registration).
 *M7 (L. 354-361) In line with Perneger (1998), we argue that applying Bonferroni corrections for a priori hypotheses has advantages and disadvantages. One of the disadvantages is that the adjusted α is often smaller than necessary, particularly if there are quite a few tests and/or the test statistics are positively correlated (all social reactions). Therefore, this method often fails to detect real differences and implies an increase in likelihood of type II-error to occur (Lee & Lee, 2018). After weighing out the (dis)advantages, we decided to not apply this conservative correction to our dependent variables that measured social reactions, to not miss possible effects and to avoid type II-errors.
(L.341-343) : The main research questions, key variables, conditions and analyses were pre-registered on AsPredicted (‘Narrative coherence and the response of others’, #9816, https://aspredicted.org/y3d8n.pdf).
(L. 497-499): Pearson correlations between the mean scores on the dependent variables over the four stories and the secondary independent variables (characteristics of the listener) were calculated and Bonferroni corrections were applied (*$\alpha$ *< .00625) since hypotheses for this part of the analyses were exploratory.*

8. With regard to the main effect of valence, I had two thoughts. First, I am not sure I would treat this as a focal main effect at all. The story/paper is not about valence. I think including it as a condition is fine, but I might suggest “downplaying” valence in the paper. Also, with respect to the findings for negative valence, the authors’ explanation seemed unnecessarily complex to me. Isn’t a simpler explanation just that people are more empathetic, etc. to those to whom they perceive need it more. Related to this, I wonder if you are picking up on intensity (instead of valence). Just a thought. . .
R8. Thank you for your comments. We do agree that valence is not the main focus of this paper, but rather a side-effect of using memory coherence and its coding in line with the tradition of investigating it in positive and negative memories separately. We explained this in the manuscript as well, stating that we only investigated it secondarily. We now also downplayed valence in the results section, by focusing on main effects on coherence, and only afterwards on how coherence can interact with valence. The explanation that people seem more empathetic to those who need it more would be not in line with our findings that incoherence, which is often interpreted as a sign of an unfinished meaning-making process, was reacted upon with less empathy and not more. Our explanations are also grounded in the interactional theory of depression, which we discuss in our manuscript.
Furthermore, we did control for intensity, by having evaluators rate the memories not only on positive or negative, but on a scale for ‘extremeness of the positivity/negativity’ going from -5 to +5. We made sure that the memories we used were as extremely positive as they were negative (scores of +4 comparable with scores of -4 in terms of intensity).

*M8(a) (L.161-163): Since memory coherence is mostly assessed in the form of narratives about high-impact positive and negative memories, valence of the memories was assessed secondarily.
 (L. 224-232): They received the instructions to read the stories closely and after each one indicate how negative or positive they consider the story to be on a scale from -5 (extremely negative) to +5 (extremely positive). We adhered to the literature here in the sense that narrative coherence is assessed by summing the score of coherence of a very positive and a very negative event. Furthermore, social sharing of emotional events can indeed involve both positive and negative events. Hence, it was important for us to investigate whether coherence of the speaker would have an overruling effect on valence in influencing reactions of the listener (for example: even though something positive is shared, it could still be rated to be unpleasant, because it was incoherently narrated upon).
 (L. 379-490):* ***Main effects of memory coherence on listener’s social reactions*** *For willingness (Fig 1A), there was a significant main effect of Coherence, F (1, 95) = 26.81, p < .001, η_p_^2^ = .22, indicating that participants were more willing to interact with those who told a coherent story compared to those who told an incoherent story, M_C_ = 34.72, SE_C_ = .65, M_IC_ = 31.45, SE_IC_ = .69.*
 *With regards to socio-emotional reactions (Fig 1B), there was a significant main effect of Coherence, F (1, 95) = 51.81, p < .001, η_p_^2^ = .35, illustrating that participants showed more empathy and trust towards those who told a coherent story compared to those who told an incoherent story, M_C_ = 40.54, SE_C_ = .51, M_IC_ = 35.64, SE_IC_ = .58.*
 *Participants had more positive feelings for those who told a coherent story compared to those who told an incoherent story, M_C_ = 4.08, SE_C_ = .09, M_IC_ = 3.76, SE_IC_ = .09, as indicated by a significant main effect of Coherence, F (1, 95) = 12.35, p = .001, η_p_^2^ = .12, on positive feelings experienced towards the other* *(Fig 1C). Findings were similar for negative feelings experienced towards the other* *(Fig 1D), as there was again a significant main effect of Coherence, F (1, 95) = 9.60, p = .003, η_p_^2^ = .09. This illustrates that participants had more negative feelings towards those who told an incoherent story compared to those who told a coherent story, M_C_ = 2.20, SE_C_ = .09, M_IC_ = 2.49, SE_IC_ = .09.*
 *For positive feelings experienced by the listener (Fig 1E) however, there was no main effect of Coherence, F (1, 95) = 1.95, p = .17, η_p_^2^ = .02.* *Regarding main effects, similar results were found for negative feelings experienced by the listener (Fig 1F). There was no main effect of Coherence, F (1, 95) = 1.42, p = .24, η_p_^2^ = .02.*
 *In line with feelings experienced by the listener, there was no main effect of Coherence, F (1, 95) = 2.55, p = .11, η_p_^2^ = .03 on emotional support (Fig 1G). Results differed from the previous, as for instrumental support (Fig 1H), there was a significant main effect of Coherence, F (1, 95) = 10.96, p = .001, η_p_^2^ = .10. Participants in fact indicated they would give more instrumental support to someone telling a coherent story compared to someone telling an incoherent story, M_C_ = 8.56, SE_C_ = .18, M_IC_ = 8.05, SE_IC_ = .21.*
***Interaction effect of coherence and valence on listener’s social reactions*** *Coherence interacted with valence to impact negative feelings experienced by the listener, F (1, 95) = 8.80, p = .004, η_p_^2^ = .09. Follow-up paired sample t-tests showed that this interaction effect was due to a difference in responding to coherence, depending on the valence of the story. Remarkably, participants experienced more negative feelings when hearing someone telling an incoherent story compared to when hearing someone telling a coherent story, but only for stories with a positive valence, t(95) = -2.93, p = .004, not for stories with a negative valence, t(95) = 1.12, p = .27. This could mean that tolerance for incoherence is lower (more negative feelings) when someone is talking about a positive event, M_CP_ = 2.13, SE_CP_ = .11, M_ICP_ = 2.47, SE_ICP_ = .12, whereas we are more forgiving towards those that talk about a negative event in an incoherent manner, M_CN_ = 3.20, SE_CN_ = .13, M_ICN_ = 3.06, SE_ICN_ = .12. This will be further explained in the discussion.
M8(b) (L460-464): Referring back to the interactional theory of depression (17), it seems that mood induction (in terms of feelings in the listener him/herself) in this study takes place more so according to the valence, and not so much according to the coherence of the memory, whereas the opposite is true for social rejection (in terms of willingness and instrumental support), which does take place towards incoherently talking people, but not towards people talking about something negative.*

*Table 1. Overview of scores of narratives on coherence and valence*

|  | *Topic* | *Coherence rating (0-9)* | *Valence rating (-5 to +5)* |
| --- | --- | --- | --- |
| *Narrative 1* | *Graduation* | *9* | *4.14* |
| *Narrative 2* | *Falling in love* | *3* | *4.14* |
| *Narrative 3* | *Suicide friend* | *9* | *-4.14* |
| *Narrative 4* | *Divorce parents* | *3* | *-4* |

I wish the authors well in this and their other research endeavors.
Thank you very much.

**Reviewer #3:** Thank you for the opportunity to review this manuscript.
The manuscript is well written and the topic is very interesting.
I would suggest some minor revisions.

The t tests for the narratives are run for very low sample, but the t tests needs at least N = 30. I would suggest the use of a nonparametric test.
Thank you for considering the topic to be interesting and for your suggestion. We decided to leave out the t-tests you mention since we considered them not to be of added value for comparing the scores described in the manuscript.

Sometimes the authors use 2 or 3 decimals... In table 1, M have 2 decimals, SD have 3 decimals.
Thank you for noticing this, we have changed this to 2 decimals everywhere.
